# The 58th Cysteine of TcpP Is Essential for *Vibrio cholerae* Virulence Factor Production and Pathogenesis

**DOI:** 10.3389/fmicb.2020.00118

**Published:** 2020-02-06

**Authors:** Mengting Shi, Na Li, Yuanyuan Xue, Zengtao Zhong, Menghua Yang

**Affiliations:** ^1^College of Life Sciences, Nanjing Agricultural University, Nanjing, China; ^2^Key Laboratory of Applied Technology on Green-Eco-Healthy Animal Husbandry of Zhejiang Province, College of Animal Science and Technology, Zhejiang A&F University, Hangzhou, China

**Keywords:** *Vibrio cholerae*, TcpP, cysteine residue, virulence gene regulation, pathogenesis

## Abstract

*Vibrio cholerae*, the causative agent of the severe diarrheal disease cholera, has evolved signal transduction systems to control the expression of virulence determinants. It was previously shown that two cysteine residues in the periplasmic domain of TcpP are important for TcpP dimerization and activation of virulence gene expression by responding to environmental signals in the small intestine such as bile salts. In the cytoplasmic domain of TcpP, there are another four cysteine residues, C19, C51, C58, and C124. In this study, the functions of these four cysteine residues were investigated and we found that only C58 is essential for TcpP dimerization and for activating virulence gene expression. To better characterize this cysteine residue, site-directed mutagenesis was performed to assess the effects on TcpP homodimerization and virulence gene activation. A TcpP_C__58__S_ mutant was unable to form homodimers and activate virulence gene expression, and did not colonize infant mice. However, a TcpP_C__19__/__51__/__124__S_ mutant was not attenuated for virulence. These results suggest that C58 of TcpP is indispensable for TcpP function and is essential for *V. cholerae* virulence factor production and pathogenesis.

## Introduction

*Vibrio cholerae* is a Gram-negative, facultative human pathogen and is the causative agent of cholera. The *Vibrio* life cycle begins with a free swimming phase in aquatic environments. Upon oral ingestion, *V. cholerae* passages through the stomach to reach the small intestine, its primary site of colonization. During its life cycle, *V. cholerae* changes its transcriptional profile in different environmental niches to facilitate survival and colonization fitness (Zhu and Mekalanos, 2003; Moorthy and Watnick, 2005; Matson et al., 2007; Nielsen et al., 2010; Mandlik et al., 2011). During the early infection, *in vivo* virulence gene expression is induced by a number of host signals, including anoxic environment and chemicals present in the small intestine (Kovacikova et al., 2010; Yang et al., 2013; Fan et al., 2014; Hay et al., 2017; Midgett et al., 2017). In late infection stages, virulence genes are repressed and a coordinated “escape response” allows the organism to detach from the intestinal surface in preparation for exit from the host (Larocque et al., 2005; Nielsen et al., 2006, 2010).

The membrane-bound transcriptional regulator TcpP plays a critical role in *V. cholerae* virulence. A TcpP partner protein, TcpH, stabilizes TcpP and enhances its activity (Krukonis et al., 2000; Crawford et al., 2003; Beck et al., 2004). TcpP, in conjugation with ToxR, activates the transcription of *toxT*, the master regulator of numerous virulence factors in *V. cholerae* (Krukonis et al., 2000; Krukonis and DiRita, 2003; Haas et al., 2015). The two cysteine residues in the periplasmic domain of TcpP are critical for its activation of *toxT* (Yang et al., 2013; Morgan et al., 2016; Xue et al., 2016). Intermolecular disulfide bond formation in TcpP results in homodimerization and the activation of virulence gene expression (Yang et al., 2013). And the formation of intramolecular disulfide bond in TcpP enhances the stability of TcpP (Morgan et al., 2016). There are another four cysteine residues in the cytoplasmic domain of TcpP, the functions of which are under investigation. The sulfur atom in the cysteine is strategically positioned in some regulatory proteins to function as a sensor of reactive oxygen species (ROS) or reactive nitrogen species (RNS) (Vazquez-Torres, 2012). AphB, the transcriptional activator of TcpP in *V. cholerae*, apply the signaling properties of thiol switches to regulate virulence and ROS relevant genes expression to increase the fitness of *V. cholerae* during its life cycle (Liu et al., 2011, 2016a,b). A growing number of Gram-positive and Gram-negative pathogens are now known to apply the signaling properties of thiol switches in order to increase fitness during their associations with vertebrate hosts (Vazquez-Torres, 2012).

The transmembrane protein TcpP contains four cysteines in the cytoplasmic domain which may play roles in regulating virulence gene expression in *V. cholerae*. We mutated one of these cysteines, C58, to serine and found that this TcpP mutant cannot homodimerize and loses transcriptional activation activity. Here we further investigated the role of TcpP C58 in the context of *V. cholerae* infection.

## Materials and Methods

### Bacterial Strains, Plasmids and Growth Conditions

Strains, plasmids, and oligonucleotides used in this study are summarized in Supplementary Table S1. All *V. cholerae* strains used in this study were derived from E1 Tor C6706 (Joelsson et al., 2006), and were propagated in LB media containing appropriate antibiotics at 37°C unless otherwise noted. Transcriptional *lux* reporters of *toxT* promoter regions in the pBBR-lux vector (Liu et al., 2011) have been described previously (Yang et al., 2013). Plasmids for overexpressing TcpP or mutants were either described previously (Yang et al., 2013) or were constructed by cloning the PCR-amplified coding regions into pBAD24 (Guzman et al., 1995) or pACYC117 (Chang and Cohen, 1978). In-frame deletion strains used in this study were described in previous publications (Liu et al., 2011; Yang et al., 2013). TcpP truncation and chimeric mutants as well as cysteine mutations were constructed by using overlap extension PCR (Higuchi et al., 1988).

### Measurement of Virulence Gene Expression and Virulence Factor Production

Overnight cultures of *V. cholerae* strains containing virulence promoter *luxCDABE* transcriptional fusions were subcultured at a dilution of 1:100 in LB with or without bile salts indicated and grown anaerobically until OD_600_ ≈ 0.2. Luminescence was measured using a Bio-Tek Synergy HT spectrophotometer and normalized for growth against OD_600_. Luminescence expression was reported as light units/OD_600_. TCP production was measured by Western blot analysis using an anti-TcpA polyclonal antibody (Zhu et al., 2002).

### TcpP Cytoplasmic Domain Expression and Purification

The DNA fragment encoding the cytoplasmic domain of TcpP (AAs 1–135) was amplified from genomic DNA. The PCR products were inserted into a modified pET-28a vector encoding an N-terminal His_6_ tag. Escherichia *coli* BL21(DE3)/pLysS cells were transformed with the plasmid containing the target gene and transformed cells were used for protein expression by autoinduction (Thompson et al., 1994). Proteins were expressed and purified on nickel columns according to the manufacturer’s instructions (Novagen).

### Gel Retardation Assay of TcpP Cytoplasmic Domain With *toxT* Promoter

His_6_ tag fused protein of TcpP_cyto_ was expressed and purified as described above. DNA fragments of the *toxT* (VC0838) promoter region were generated using PCR and purified with QIAquick PCR Purification Kit (Qiagen) according to the manufacturer’s instructions. DNA (50 ng) was incubated with varying concentrations of purified protein and incubated in binding buffer (50 mM Tris–HCl, pH 8.0, 250 mM NaCl, 5.0 mM MgCl_2_, 2.5 mM DTT, 2.5 mM EDTA and 20% glycerol) for 30 min at room temperature. Protein-DNA complexes were separated electrophoretically on a native 5% polyacrylamide gel at 100 V in 0.5 × Tris-acetate-EDTA (TAE) buffer and visualized using ethidium bromide staining.

### *In vitro* Redox State Determination of TcpP Cytoplasmic Domain

The *in vitro* redox state of TcpP cytoplasmic domain was assessed using AMS trapping experiments as (Denoncin et al., 2013). 100 ng of freshly purified TcpP_cyto_ protein was precipitated by 10% (vol/vol) trichloroacetic acid (TCA). Precipitated proteins were washed three times with cold acetone, suspended in a buffered solution containing 100 mM Tris–HCl pH 7.5 and 1% (wt/vol) SDS, with or without 10 mg/mL AMS, and incubated in the dark at 30°C for 30 min followed by 37°C for 10 min. AMS alkylation was stopped by the addition of SDS loading buffer [2% (wt/vol) SDS, 50 mM Tris, 10% (vol/vol) Glycerol, 142 mM 2-mercaptoethanol]. Proteins were separated by SDS-PAGE, and immunoblot analyses were performed.

### Bacterial Two-Hybrid System to Determine TcpP Mutant Interaction

To analyze the dimerization of TcpP mutant, β-galactosidase measurements were performed as described previously (Yang et al., 2013). Briefly, overnight cultures of *E. coli* BTH101 containing both pUT-18C-fusion and pKT25-fusion constructs (Karimova et al., 1998) were subcultured at a dilution of 1:100 in LB medium containing 0.5 mM isopropyl-β-D-1-thiogalactopyranoside and incubated without shaking at 30°C for 8 h. Cultures were then assayed for β-galactosidase activity (Karimova et al., 1998).

### Differential Thiol Trapping Assay of Disulfide Bond

The thiol/disulfide state of the cytoplasmic cysteines of TcpP was monitored *in vivo* by differential thiol trapping (Tetsch et al., 2011). *V. cholerae*Δ*tcpPH* strains carrying the plasmid pacyc-TcpP_C__207__/__218__S_-cFLAG or P*_BAD_*-TcpP_C__58__S_-nFLAG were grown in LB medium to an OD_600_ of about 0.5. Subsequently, overproduction of the TcpP mutant-FLAG derivatives was induced by addition of 0.1% (w/v) arabinose for 5 min. Bacterial cells were collected and resuspended in PBS containing 100 mM iodoacetamide, and then incubated at 37°C with 1,300 rpm agitation for 1 h. This first alkylation procedure irreversibly modified all free thiol groups. Subsequently, cells were harvested and lysed in 1 mL ice-cold 10% (w/v) TCA and stored on ice for at least 6 h or at 4°C overnight. The TCA treated cells were centrifuged (16,000 *g*, 4°C, 15 min), and the resulting pellet was washed twice with 500 μl of ice-cold acetone. The supernatant was removed and the pellet was resuspended in 900 μl of 100 mM Tris–HCl (pH 7.5), 1% (w/v) SDS supplemented with 50 mM DTT to reduce disulfide bonds. After 1 h of incubation in the dark (37°C, gentle agitation at 1,300 rpm), 100 μl ice-cold 100% (w/v) TCA was added, and the sample was stored on ice for at least 6 h. After centrifugation, the resulting pellet was again washed twice with 500 μl of ice-cold acetone. Finally, the pellet was resuspended in 50 μl of Tris-SDS buffer, with or without 10 mM AMS, and incubated at 30°C for 30 min followed by 37°C for 10 min. AMS alkylation was stopped by the addition of SDS loading buffer. Proteins were separated by SDS-PAGE, and immunoblot analyses were performed. TcpP was detected using a monoclonal anti-FLAG antibody(Sigma–Aldrich).

### *In vivo* Competition Assays

All animal experiments were carried out in strict accordance with the animal protocols that were approved by the Institutional Animal Care and Use Committee of Zhejiang A&F University (Permit Number: ZJAFU/IACUC_2011-10-25-02). Five-days-old ICR mice purchased from the animal facility of Zhejiang Academy of Medical Sciences were raised in the animal facility of Zhejiang A&F University and separated from their dams 1 h before infection. Subsequently, they were anesthetized by inhalation of isoflurane gas and then inoculated by oral gavage with 50 μL of an appropriate dilution of the 1:1 mixture (WT and mutant), resulting in an infection dose of ∼10^6^ cfu per mouse. To determine the exact inputs, appropriate dilutions of the inocula were plated on LB-Sm/X-Gal plates. After 22 h, the mice were euthanized, and the small intestines from each mouse were collected by dissection. The small intestines were mechanically homogenized in LB broth with 15% glycerol, and appropriate dilutions were plated on LB-Sm/X-Gal. The competitive index was calculated as the ratio of mutant to wild type colonies normalized to the input ratio.

## Results

### TcpP C58 Is Indispensable for Activating Virulence Gene Expression

**FIGURE 1:**
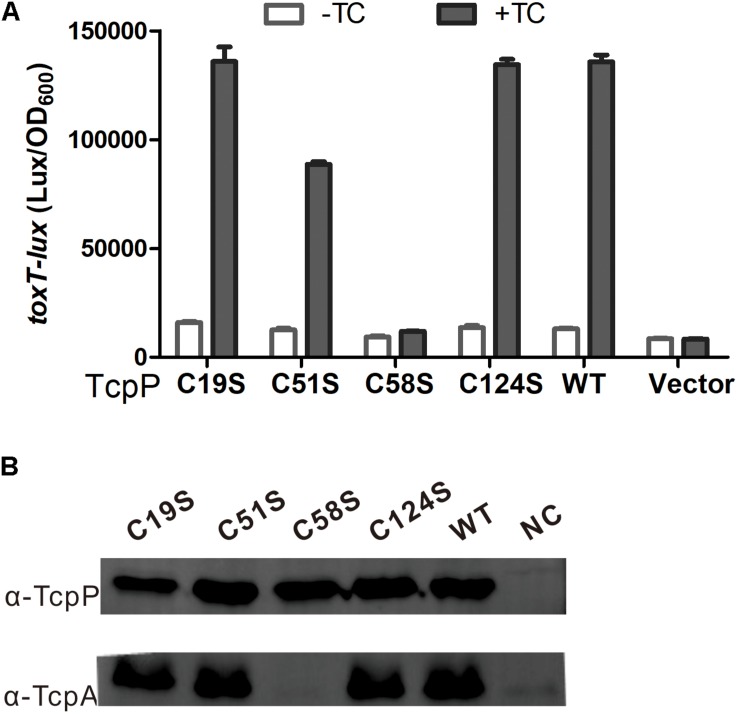
C58 is indispensable for TcpP activation of virulence gene expression. **(A)** *V. cholerae* Δ*tcpP* strains containing P*_BAD_*-controlled plasmids harboring TcpP or its cysteine mutant derivatives and a P*_toxT_-lux* transcriptional fusion plasmid were grown in LB with 0.01% arabinose in the presence or absence of taurocholate acid at 37°C until OD600 ≈ 0.2. Luminescence was measured and reported as light units/OD_600_. Data are the means ± SD (*n* = 3). **(B)** *V. cholerae*Δ*tcpP* strains containing P*_BAD_*-TcpP wild type or cysteine mutant plasmids grown in LB with 0.01% arabinose in the presence of 1 mM TC for 6 h. Cell lysates (0.1 mg) were applied to SDS-PAGE, and subjected to Western blotting using an anti-TcpP or anti-TcpA antibody.

Previous studies showed that the two cysteines (C207 and C218) in the periplasmic domain of TcpP are crucial for TcpP activity (Yang et al., 2013). Bile salts induce TcpP to form an intermolecular disulfide bond between these two cysteines to dimerize by interfering with the redox potential of the Dsb proteins (Yang et al., 2013; Xue et al., 2016). In the cytoplasmic domain of TcpP, there are four additional cysteines C19, C51, C58, and C124. To study if these four cysteines are also indispensable for TcpP activity, we mutagenized these four cysteines to serines respectively and analyzed their activities by quantifying *toxT* transcription. We found that TcpP_C__19__S_, TcpP_C__51__S_, and TcpP_C__124__S_ activated *toxT* expression to the same level as TcpP wild type (TcpP_WT_), but TcpP_C__58__S_ did not (Figure 1). The TcpP_C__58__S_ mutant was still localized to the bacterial membrane (Supplementary Figure S1).

### TcpP_C__58__S_ Cyto-Domain Cannot Bind DNA *in vitro*

**FIGURE 2:**
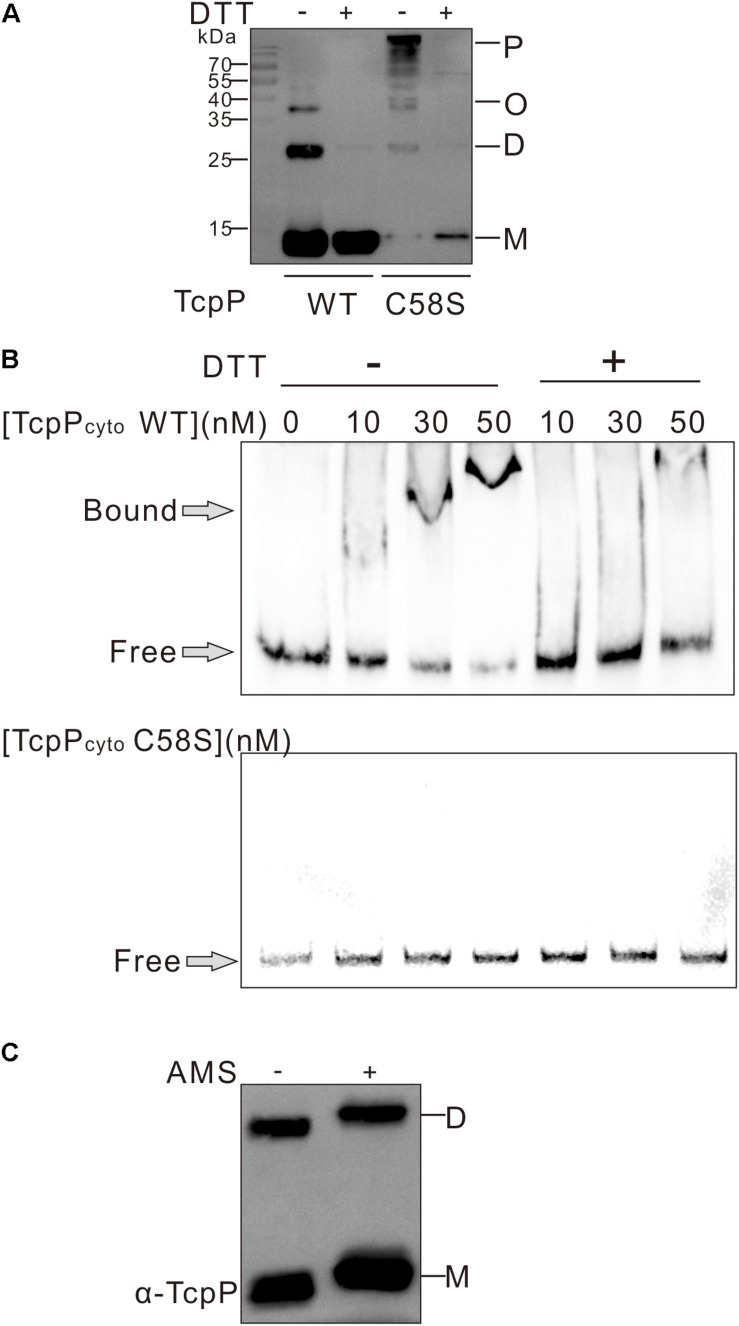
TcpP cytoplasmic domain binding to DNA *in vitro*. **(A)** The cytoplasmic domain of TcpP WT or C58S including the first 135 amino acids of the N termini was purified under non-reducing conditions. Proteins treated with (+) or without (–) DTT were separated using SDS-PAGE and subjected to Western blotting using an anti-TcpP antibody. P, polymer; O, oligomer; D, dimer; M, monomer. **(B)** Gel shift assays using purified TcpP_cyto_ WT or C58S and DNA containing ∼400 bp of the regulatory regions identified in *toxT* promoter. **(C)** Free thiol group assay of TcpP_cyto_ WT protein by AMS trapping. One hundred ng of protein was precipitated using 10% TCA and proteins containing free thiol groups were trapped with AMS. TcpP_cyto_ was detected by Western blot using anti-TcpP antibody.

The cytoplasmic domain of TcpP contains an N-terminal winged helix-turn-helix (wHTH) DNA binding domain of the OmpR family (Martinez-Hackert and Stock, 1997; Krukonis and DiRita, 2003). To determine if the C58S mutation affects TcpP binding to DNA, we performed gel retardation assays of TcpP_cyto_ WT or TcpP_cyto_ C58S, which each contain the N-terminal 135 amino acids of TcpP. Both the cytoplasmic domain of TcpP_cyto_ WT and TcpP_cyto_ C58S could be expressed and purified as soluble protein. However, sodium dodecyl sulfate-polyacrylamide gel electrophoresis (SDS-PAGE) of these two purified proteins showed that under non-reducing conditions, TcpP_cyto_ C58S formed oligomers or polymers, while TcpP_cyto_ WT mainly exists as monomer or dimer (Figure 2A). Under non-reducing conditions TcpP_cyto_ WT bound *toxT* promoter DNA as a function of increasing protein concentrations, while TcpP_cyto_ C58S did not bind DNA under the same conditions (Figure 2B).

The lack of DNA binding by TcpP_cyto_ C58S suggested that TcpP_cyto_ binding to DNA requires disulfide bond formation. By analyzing the redox status TcpP_cyto_ WT, we found that TcpP_cyto_ WT is not totally oxidized but still contains free thiol groups either in the monomer or the dimer form of the protein (Figure 2C). This might indicate that proper disulfide bond forming could help TcpP_cyto_ WT folding and DNA binding.

### C58 Is Indispensable for TcpP Homodimerization

**FIGURE 3:**
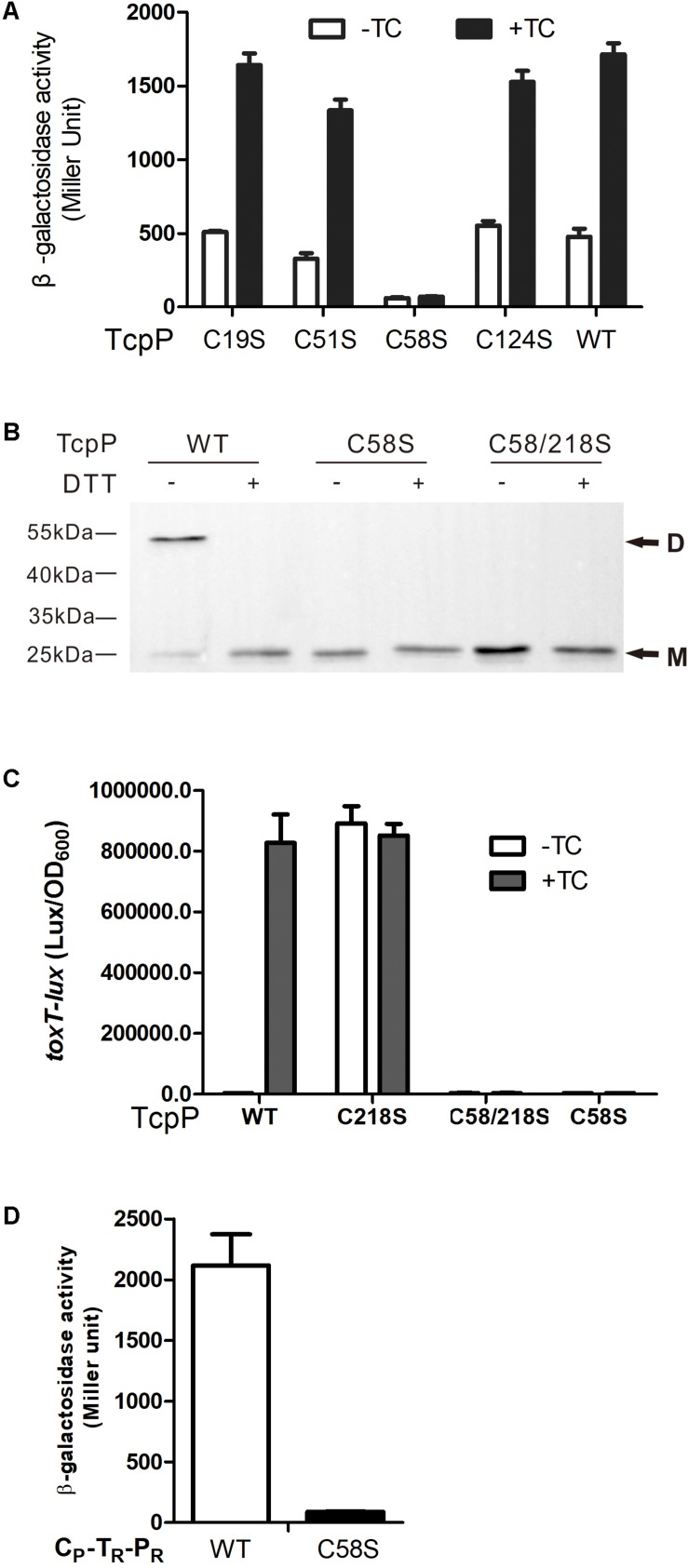
C58 of TcpP is indispensable for protein dimerization. **(A)** Full-length TcpP wild type or its cysteine mutant was fused with the T25 and T18 domains of adenylate cyclase (CyaA) from *Bordetella pertussis*, respectively, and the T25, T18 fusion pairs were introduced into *E. coli cyaA* mutants (Karimova et al., 1998). Cultures were grown at 30°C for 8 h without shaking and β-galactosidase activity was measured and reported as Miller Units (Miller, 1972). Data are means ± SD (*n* = 3). **(B)** *V. cholerae* Δ*tcpP* containing P*_BAD_*-controlled plasmids harboring TcpP or its cysteine mutant derivatives fused with N-terminal FLAG tags were grown in LB containing 0.01% arabinose in the presence of taurocholate acid at 37°C for 6 h. Cell lysates (0.1 mg) were applied to SDS-PAGE with (+) or without (–) 50 mM of DTT, and subjected to Western blotting using an anti-FLAG antibody. D, dimer; M, monomer. **(C)** *E. coli* DH5a strains containing P*_BAD_*-controlled plasmids harboring TcpP or its cysteine mutant derivatives and a P*_toxT_-lux* transcriptional fusion plasmid were grown in LB with 0.01% arabinose in the presence or absence of taurocholate acid at 37°C until OD600 ≈ 0.2. Luminescence was measured and reported as light units/OD_600_. Data are the means ± SD (*n* = 3). **(D)** Chimeric TcpP was fused with the T25 and T18 domains and the T25, T18 fusion pairs were introduced into *E. coli cyaA* mutants (Karimova et al., 1998). Cultures were grown at 30°C for 8 h without shaking and β-galactosidase activity was measured and reported as Miller Units (Miller, 1972). Data are means ± SD (*n* = 3).

We previously found that TcpP_C__218__S_ which can constitutively form homodimer activates *toxT* expression in the absence of bile salts, while TcpP_C__207__S_ losing the ability of homodimerization cannot activate *toxT* expression (Yang et al., 2013). We tested the hypothesis that the TcpP_C__58__S_ mutant cannot homodimerize by performing bacterial two-hybrid and protein immunoblotting studies. TcpP_C__58__S_ failed to form homodimers in both assays (Figures 3A,B). TcpP is an inner-membrane protein of *V. cholerae*. To test if mutagenizing C218S can recover TcpP_C__58__S_ activity, we constructed a TcpP_C__58__/__218__S_ mutant. However, like TcpP_C__58__S_, TcpP_C__58__/__218__S_ failed to activate *toxT* expression (Figure 3C) and did not form dimers (Figure 3B). By testing the dimerization of the chimeric protein of TcpP and ToxR (C_P_-T_R_-P_R_), another inner-membrane protein of *V. cholerae* and also a master regulator of virulence gene expression, we also found that the chimeric protein can form homodimers, but when the 58th cysteine of TcpP was substituted to serine, the mutant could not dimerize (Figure 3D). These results indicated that the cytoplasmic domain of TcpP plays an important role in protein homodimerization and activity.

### TcpP Cytoplasmic Domain Contains No Disulfide Bonds

**FIGURE 4:**
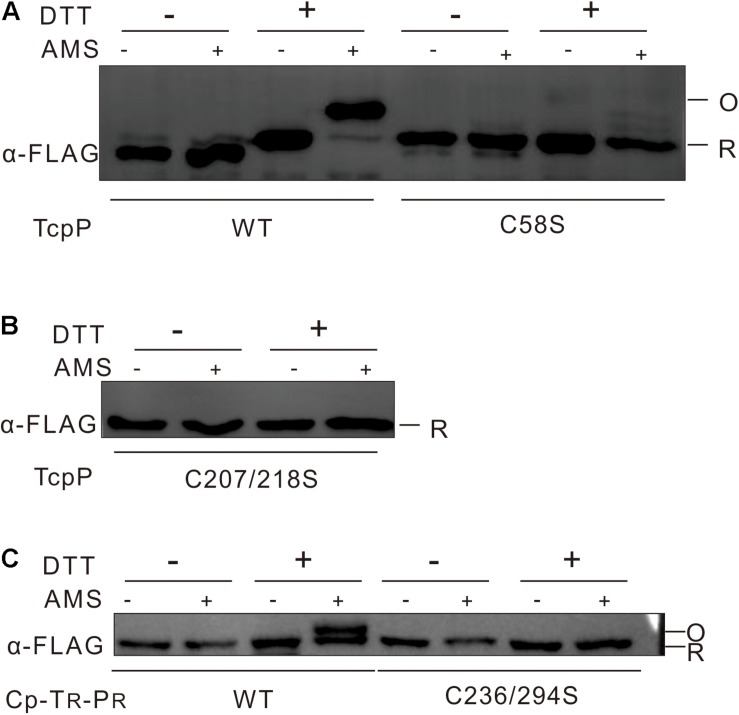
No disulfide bond could be detected in TcpP cytoplasmic domain. **(A)** *V. cholerae* Δ*tcpP* containing P*_BAD_*-controlled plasmids harboring TcpP or its cysteine mutant derivatives fused with C-terminal FLAG tags were incubated at 37°C until OD600 ≈ 0.5. To label free thiol groups irreversibly, 100 mM iodoacetamide was added directly to the living cells. After TCA precipitation and extensive washing, oxidized thiol groups were reduced by addition of 50 mM DTT (+) in denaturing buffer or not (–). These reduced cysteines were then alkylated by addition of 10 mM AMS (+) or not (–). Samples were mixed with non-reducing SDS-sample buffer and loaded onto 12.5% SDS-polyacrylamide gels. TcpP was detected by Western blot analysis of the FLAG-tagged proteins. Blot shown is representative of at least three separate experiments. **(B)** Disulfide bond assay of *V. cholerae* Δ*tcpP* strain containing P*_BAD_*-controlled plasmids harboring TcpP_C__207__/__218__S_ was used the same methods as described in panel A. **(C)** Disulfide bond assay of *E. coli* strain containing chimeric TcpP was used the same methods as described in panel A.

We next wanted to determine whether TcpP needs to form disulfide bonds between C58 to activate virulence gene expression in *V. cholerae*. We first tested the disulfide bond formation of TcpP_WT_ and TcpP_C__58__S_ by performing AMS labeling assay. We found that TcpP_WT_ contains disulfide bonds, but could not use this information to determine whether disulfide bonds form between the cysteines in the cytoplasmic domain, because it is very likely that disulfide bonds formed between C207 and C218 would be oxidized by DsbA in the periplasm (Xue et al., 2016). Surprisingly, no disulfide bonds were detected in the TcpP_C__58__S_ mutant (Figure 4A), indicating that the mutagenesis of C58 not only changes the conformation of the TcpP cytoplasmic domain but also the periplasmic domain.

To detect specifically disulfide bond formation in the cytoplasmic domain of TcpP, we then tested the disulfide bond formation of TcpP_C__207__/__218__S_ mutant by the same methods. No disulfide bond formation could be observed in this case (Figure 4B). To determine whether disulfide bonds form between C58 residues when TcpP dimerizes, we created a chimeric protein consisting of the cytoplasmic domain of TcpP and the transmembrane and periplasmic domains of ToxR (C_P_-T_R_-P_R_) with both of the 236th and 294th cysteine of ToxR substituted to serine. This chimeric protein activated *toxT* transcription and formed homodimers (Supplementary Figure S2). This chimeric protein did not form disulfide bonds (Figure 4C), suggesting that the disulfide bonds formed in TcpP_WT_ are likely between C207 and C218, and that it is unlikely that disulfide bonds form in the cytoplasmic domain of TcpP.

### TcpP_C__58__A_ and TcpP_C__58__T_ Can Activate Virulence Gene Expression

**FIGURE 5:**
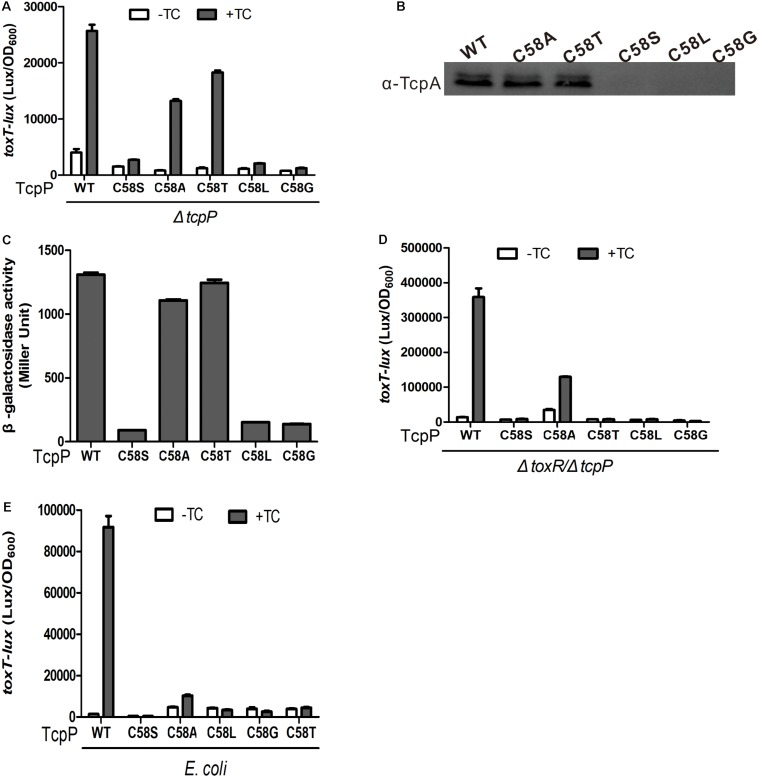
TcpP mutants activity and homodimerization assay. **(A)** *V. cholerae* Δ*tcpP* containing P*_BAD_*-controlled plasmids harboring TcpP or its cysteine mutant derivatives were grown in LB with 0.01% arabinose in the presence or absence of 1 mM TC at 37°C until OD600 ≈ 0.2. Luminescence was measured and reported as light units/OD_600_. Data are the means ± SD (*n* = 3). **(B)** *V. cholerae* Δ*tcpP* containing P*_BAD_*-controlled plasmids harboring TcpP or its cysteine mutant derivatives were grown in LB with 0.01% arabinose in the presence of 1 mM TC for 6 h. Then, 0.1-mg cell lysates were applied to SDS-PAGE and subjected to Western blotting using an anti-TcpA antibody. **(C)** Full-length TcpP wild type or cysteine mutants were fused with the T25 and T18 domains of adenylate cyclase (CyaA) from *B. pertussis*, respectively, and the T25, T18 fusion pairs were introduced into *E. coli cyaA* mutants (Karimova et al., 1998). Cultures were grown at 30°C for 8 h without shaking and β-galactosidase activity was measured and reported as Miller Units (Miller, 1972). Data are means ± SD (*n* = 3). **(D)** P*_BAD_*-controlled plasmids harboring TcpP or its cysteine mutant derivatives and a P*_toxT_-lux* transcriptional fusion plasmid in *V. cholerae* Δ*toxR/*Δ*tcpP* **(D)** or *E. coli* DH5α **(E)** strains were grown in LB with 0.01% arabinose in the presence or absence of 1 mM TC at 37°C until OD600 ≈ 0.2. Luminescence was measured and reported as light units/OD_600_. Data are the means ± SD (*n* = 3).

To further investigate the characteristics of the 58th cysteine of TcpP, we constructed four additional TcpP mutants in which the 58th cysteine was substituted to glycine (G), alanine (A), leucine (L), or threonine (T). We found that both TcpP_C__58__A_ and TcpP_C__58__T_ activated virulence gene expression to the same level as TcpP_WT_ (Figure 5A), while TcpP_C__58__G_ and TcpP_C__58__L_, similar to TcpP_C__58__S_, lost activity (Figures 5A,B). As expected, the mutagenesis of C58A or C58T of TcpP had no effect on protein homodimerization (Figure 5C). However, in *V. cholerae toxR* and *tcpP* double knock-out mutant or in *E. coli*, these TcpP mutants did not activate *toxT* expression (Figures 5D,E), indicating that the efficiency of activating *toxT* expression decreases and is more dependent on ToxR. Thus, although dimerization of TcpP does not require inter- or intra-molecular disulfide bonds in the cytoplasmic domain, the 58th cysteine residue is essential for TcpP to function properly.

### TcpP_C__19__/__51__/__124__S_ Activates Virulence Gene Expression the Same as TcpP_WT_

**FIGURE 6:**
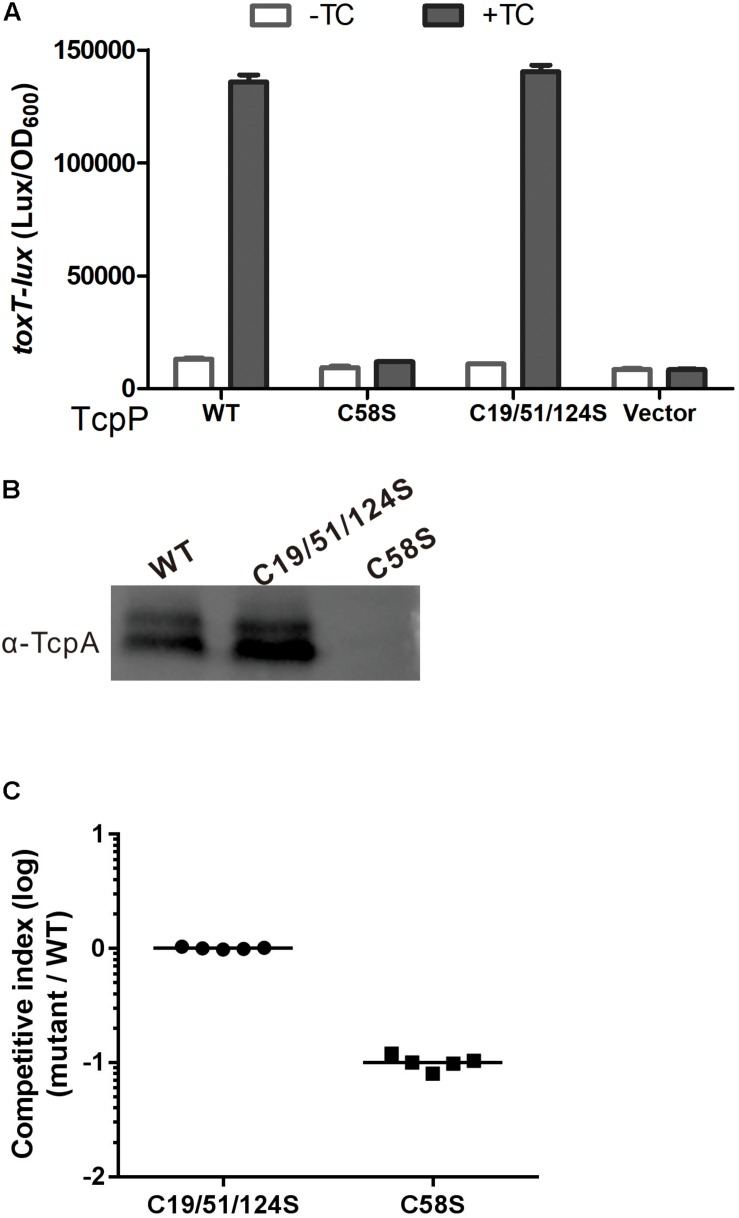
TcpP_C__19__/__51__/__124__S_ activates virulence gene expression similarly to TcpP_WT_. **(A)** *V. cholerae* Δ*tcpP* strains containing P*_BAD_*-controlled plasmids harboring TcpP or its cysteine mutant derivatives and a P*_toxT_-lux* transcriptional fusion plasmid and P*_BAD_* vector control were grown in LB with 0.01% arabinose in the presence or absence of taurocholate acid at 37°C until OD600 ≈ 0.2. Luminescence was measured and reported as light units/OD_600_. Data are the means ± SD (*n* = 3). **(B)** *V. cholerae*Δ*tcpP* strains containing P*_BAD_*-TcpP wild type or cysteine mutant plasmids grown in LB with 0.01% arabinose in the presence of taurocholate acid for 6 h. Cell lysates (0.1 mg) were applied to SDS-PAGE and subjected to Western blotting using an anti-TcpP or anti-TcpA antibody. **(C)** *In vivo* competition assay using an infant mouse model. Five-days-old ICR infant mice were inoculated with the mixture of TcpP cysteine mutant and WT at 1:1 ratio. Intestinal homogenates were collected 22 h later, and the ratio of mutant-to-WT bacteria was determined and normalized against input ratios.

To test if C58 alone is sufficient for TcpP to activate virulence gene expression, we constructed a TcpP_C__19__/__51__/__124__S_ mutant. We found that TcpP_C__19__/__51__/__124__S_ can activate virulence gene expression and form homodimers in the presence of bile salts similarly to TcpP_WT_ (Figures 6A,B).

The infant mouse model is the most commonly used animal model to study the infection and colonization of *V. cholerae* in the mammal intestine (Matson, 2018). The competition assays applied in this model, in which both a mutant and a wild type *V. cholerae* strain are infected to the same mouse and their colonization would be compared, are often used to identify factors that contribute to the virulence of *V. cholerae* (Angelichio et al., 1999; Matson, 2018). By using the infant mouse model to do the competitive experiment, we found that TcpP_C__19__/__51__/__124__S_ and TcpP_WT_ showed similar colonization efficiency, while TcpP_C__58__S_ was significantly attenuated (Figure 6C). These results indicate that C58 alone is sufficient for TcpP to activate virulence gene expression and plays an important role for *V. cholerae* to colonize in the infant mouse intestine.

## Discussion

*Vibrio cholerae*, which resides in aquatic environments as well as human intestines during its infection cycle, coordinates the transcription of virulence genes by sensing different environmental signals (Krukonis and DiRita, 2003; Matson et al., 2007). We previously reported that TcpP senses bile salt signals in the small intestine through the two cysteines in its periplasmic domain to activate the ToxT regulon (Yang et al., 2013; Xue et al., 2016). And Morgan et al. (2016) found that the formation of an intramolecular periplasmic disulfide bond in TcpP protects TcpP and TcpH from degradation in *V. cholerae*. In this study, we further investigated the functions of the cysteines in the cytoplasmic domain of TcpP, and we found that C58 is essential for TcpP homodimerization and activating virulence gene expression.

**FIGURE 7:**
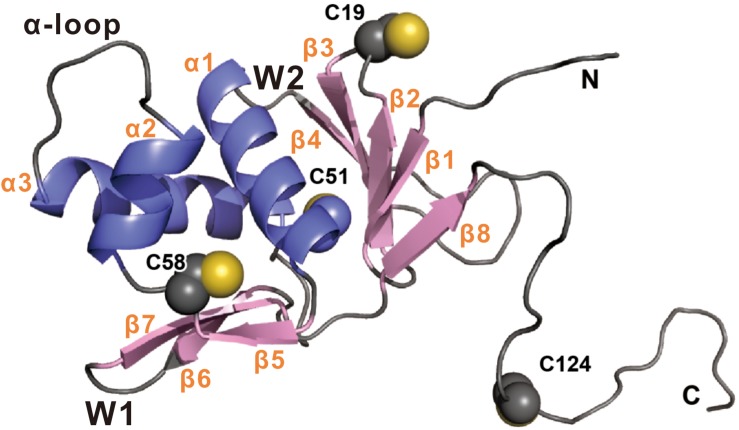
Homology model of TcpP_1__–__135_ was built using the online intFold server (McGuffin et al., 2019). The structure is shown in cartoon representation with β strand and α helix colored pink and blue, respectively. The side-chains of Cys19, Cys51, Cys58, and Cys124 are shown as spheres. Secondary structural and biologically relevant elements are labeled. α, α helix; β, β strand; W, wing.

*Vibrio cholerae* virulence is controlled by a regulatory pathway that responds to environmental signals (Matson et al., 2007). Under anaerobic conditions, TcpP is synthesized and induced by bile salts to mediate derepression and activation of ToxT, together with another membrane localized activator, ToxR (Krukonis and DiRita, 2003; Liu et al., 2011; Yang et al., 2013). Both TcpP and ToxR are members of OmpR/PhoB wHTH transcriptional activator family, and secondary structure predictions suggest that they share similar structural organization in their N termini (Martinez-Hackert and Stock, 1997; Figure 7). There is no structure available for TcpP, so we built a homology model based on the crystal structure of PhoB (Okamura et al., 2000; Figure 7). According to this model, the residue of C19 is located in the second β turn of the N-terminal β-sheet, while C51 and C58 flank the β5 strand that connects helices α1 and α2 (Figure 7). The C-terminus of TcpP_115__–__140_ is predicted to be disordered. Therefore, the position of C124 cannot be precisely mapped. This model shows that the side chain of C58 is facing inwards toward the structural core formed by three helices in the cytoplasmic domain of TcpP (Figure 7). We speculate that mutation of C58 may cause perturbation of the overall fold or dynamics of the TcpP monomer, which, in turn, may have an impact on the dimer formation of TcpP. We found that the cytoplasmic domain of TcpP mutant C58S cannot fold properly or bind to DNA *in vitro* (Figure 2). Mutating C58 to serine also prevented disulfide bond formation between the periplasmic C207 and C218 residues (Figure 3A). This indicates that the conformation of the cytoplasmic domain of TcpP can affect the folding of the periplasmic domain. Mutating C58 to alanine or threonine might not affect the structure of TcpP as significantly as that to serine, leucine or glycine. So the TcpP mutants C58S, C58L, and C58G were inactive, irrespective of the presence of ToxR, either, while C58A and C58T exhibited near wild type activity when coexpressed with ToxR (Figure 5). However, we found no evidence suggesting that disulfide bonds are required to form between C58 to promote TcpP homodimerization. Future structural studies may be needed to understand better this phenotype.

Another *Vibrio* species, *Vibrio parahaemolyticus* also possesses a TcpP homolog, VtrA, which regulates the expression of Vp-PAI genes by directly responding to bile salt concentrations (Li et al., 2016). Unlike TcpP, VtrA has only one cysteine residue in its cytoplasmic domain, C43; yet, we found that like TcpP C58S, the VtrA C43S mutant also failed to homodimerize and is unable to activate the expression of downstream virulence genes (Supplementary Figure S3). This might indicate that the cysteine residue of C58 in TcpP and C43 in VtrA regulate protein function by the same mechanism. Although the other three cysteine residues (C19, C51, and C124) were not found to play any role in TcpP homodimerization and virulence gene activation (Figure 6), we did find that these three cysteines play an important role for *V. cholerae* to survive in other environmental niches.

## Data Availability Statement

The raw data supporting the conclusions of this article will be made available by the authors, without undue reservation, to any qualified researcher.

## Ethics Statement

The animal study was reviewed and approved by the Institutional Animal Care and Use Committee of Zhejiang A&F University (Permit Number: ZJAFU/IACUC_2011-10-25-02). Written informed consent was obtained from the owners for the participation of their animals in this study.

## Author Contributions

MS, NL, and YX performed the research (the acquisition, analysis, or interpretation of the data). MS, NL, YX, ZZ, and MY analyzed the data. ZZ and MY designed the research and wrote the manuscript.

## Conflict of Interest

The authors declare that the research was conducted in the absence of any commercial or financial relationships that could be construed as a potential conflict of interest.
