# Supplementary material for: The 58th Cysteine of TcpP Is Essential for Vibrio cholerae Virulence Factor Production and Pathogenesis
Source: Front Microbiol. 2020 Feb 6;11:118. doi: 10.3389/fmicb.2020.00118 (PMC7017273; doi:10.3389/fmicb.2020.00118)
Supplement: Supplementary file 1 [file Table_1.docx]

**Supplementary Information**

**
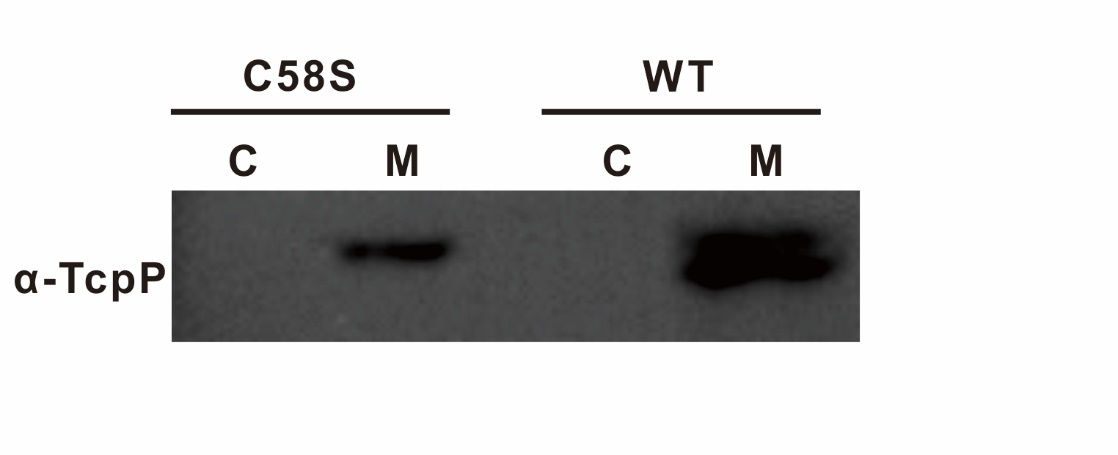
**

**Figure S1.** TcpP_WT_ and TcpP_C58S_ expression in Δ*tcpP* was induced using 0.1 % arabinose for 4 hours. Samples were then ultracentrifuged to separate the cytoplamic (C) and the membrane (M) fractions. TcpP was detected by using Western blotting with an anti-TcpP antibody.


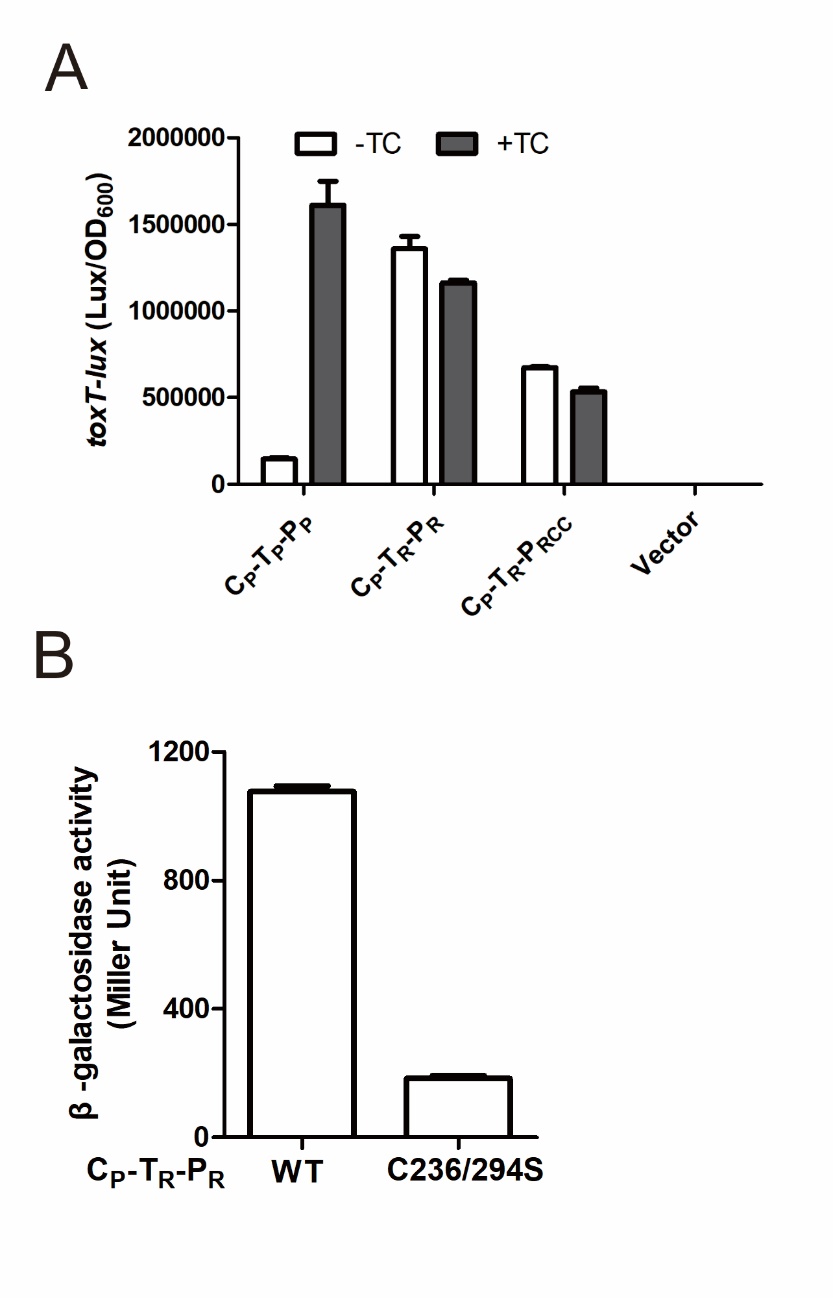


**Figure S2. A.** *E. coli* strains containing P*_BAD_*-controlled plasmids harboring full-length TcpP or chimeric TcpP and a P*_toxT_-lux* transcriptional fusion plasmid were grown in LB with 0.01 % arabinose in the presence or absence of 1 mM TC at 37 °C until OD600≈ 0.2. Luminescence was measured and reported as light units/OD_600_. Data are the means + SD (n = 3). **B.** Chimeric TcpP wild type or cysteine mutant was fused with the T25 and T18 domains of adenylate cyclase (CyaA) from *Bordetella pertussis*, respectively, and the T25, T18 fusion pairs were introduced into *E. coli cyaA* mutants(Karimova et al., 1998). Cultures were grown at 30 °C for 8 h without shaking and β-galactosidase activity was measured and reported as Miller Units (Miller, 1972). Data are means + SD (n = 3).


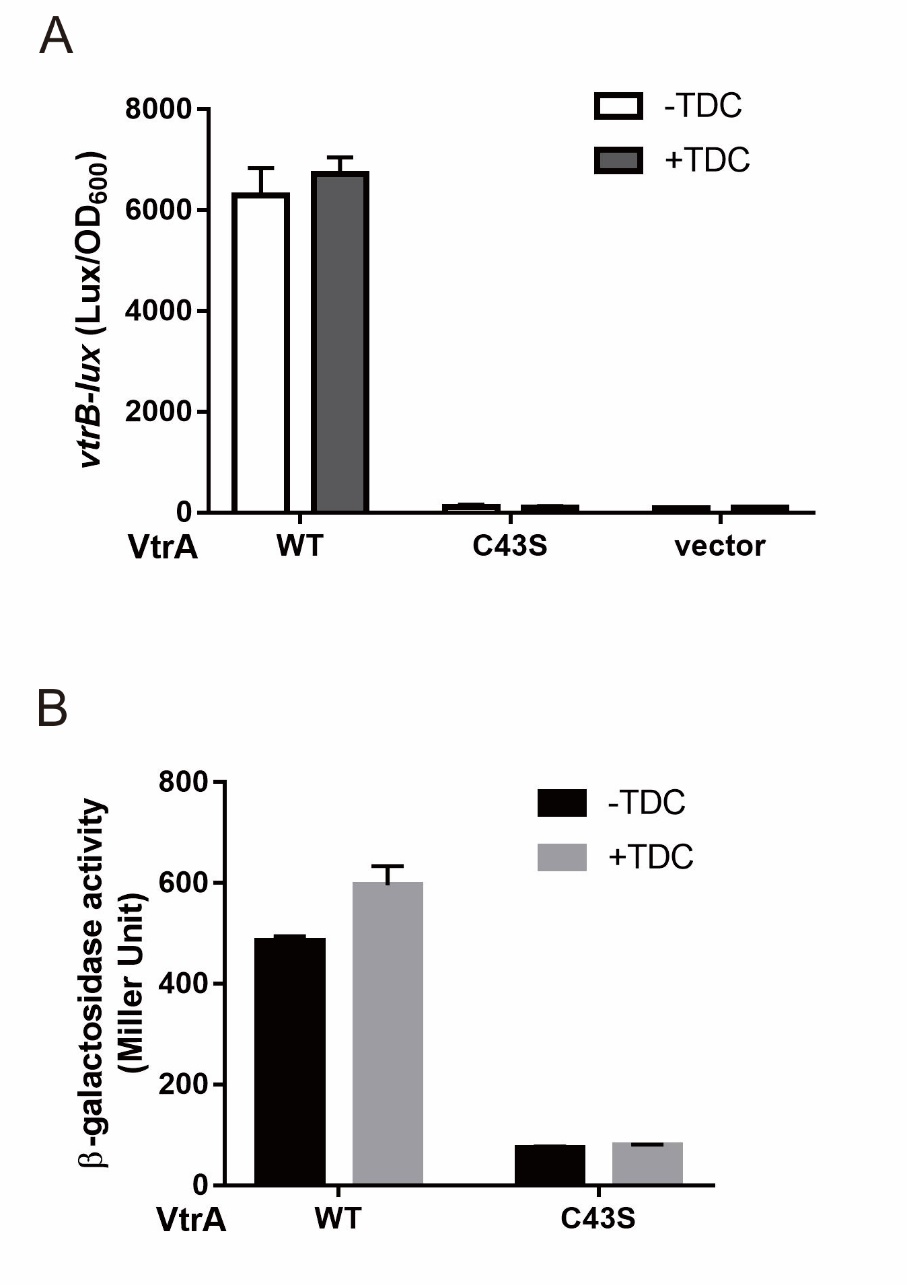


**Figure S3. A.** *E. coli* strains containing P*_BAD_*-controlled plasmids harboring VtrA wild type (WT) or cysteine mutant (C43S) and a P*_vtrB_-lux* transcriptional fusion plasmid were grown in LB with 0.01 % arabinose in the presence or absence of 1 mM taurodeoxycholate acid (TDC) at 37 °C until OD600≈0.2. Luminescence was measured and reported as light units/OD_600_. Data are the means + SD (n = 3). **B.** VtrA WT or C43S was fused with the T25 and T18 domains of adenylate cyclase (CyaA) from *Bordetella pertussis*, respectively, and the T25, T18 fusion pairs were introduced into *E. coli cyaA* mutants(Karimova et al., 1998). Cultures were grown at 30 °C in the presence or absence of 1 mM TDC for 8 h without shaking and β-galactosidase activity was measured and reported as Miller Units (Miller, 1972). Data are means + SD (n = 3).

**Table S1. Bacterial strains and primers used in this study.**

| **Strain** | **Relevant Characteristics** | | | | **Reference/Source** |
| --- | --- | --- | --- | --- | --- |
| *V.cholerae* strains |  | | | |  |
| C6706 Str^R^ | El Tor, streptomycine^R^ | | | | (Joelsson et al., 2006) |
| MHV20 | Δ*tcpP, tcpP* deletion | | | | (Yang et al., 2013) |
| MHV21 | Δ*tcpP/toxR, tcpP* and *toxR* deletion | | | | (Yang et al., 2013) |
| NLV2 | P*_tcpP_*-*tcpP_C19/51/124S_* integrated into the *lacZ* locus of Δ*tcpP* | | | | This study |
| NLV3 | P*_tcpP_*-*tcpP_C58S_* integrated into the *lacZ* locus of Δ*tcpP* | | | | This study |
| NLV9 | Δ*tcpP,* (pBAD24) , P*_toxT_-luxCDABE* | | | | This study |
| NLV10 | Δ*tcpP,* pMH501 (pBAD24, P*_BAD_-tcpP*_WT_), P*_toxT_-luxCDABE* | | | | This study |
| NLV11 | Δ*tcpP,* pNL1 (pBAD24, P*_BAD_-tcpP*_C19S_) , P*_toxT_-luxCDABE* | | | | This study |
| NLV12 | Δ*tcpP,* pNL2 (pBAD24, P*_BAD_-tcpP*_C51S_) , P*_toxT_-luxCDABE* | | | | This study |
| NLV13 | Δ*tcpP,* pNL3 (pBAD24, P*_BAD_-tcpP*_C58S_) , P*_toxT_-luxCDABE* | | | | This study |
| NLV14 | Δ*tcpP,* pNL4 (pBAD24, P*_BAD_-tcpP*_C124S_) , P*_toxT_-luxCDABE* | | | | This study |
| NLV16 | Δ*tcpP,* pMH511 (pBAD24, P*_BAD_-tcpP*_WT_-nFLAG) | | | | This study |
| NLV17 | Δ*tcpP,* pNL8 (pBAD24, P*_BAD_-tcpP*_C58S_-nFLAG) | | | | This study |
| NLV20 | Δ*tcpP/*Δ*toxR,* pNL12 (pBAD24, P*_BAD_-tcpP*_c_-*toxR_TP_*-nFLAG, WT) | | | | This study |
| NLV21 | Δ*tcpP/*Δ*toxR,* pNL13 (pBAD24, P*_BAD_-tcpP*_c_-*toxR_TP_*-nFLAG, C58S) | | | | This study |
| NLV22 | Δ*tcpP,* pNL14 (pBAD24, P*_BAD_-tcpP*_C58A_) , P*_toxT_-luxCDABE* | | | | This study |
| NLV23 | Δ*tcpP,* pNL15 (pBAD24, P*_BAD_-tcpP*_C58T_) , P*_toxT_-luxCDABE* | | | | This study |
| NLV24 | Δ*tcpP,* pNL16 (pBAD24, P*_BAD_-tcpP*_C58G_) , P*_toxT_-luxCDABE* | | | | This study |
| NLV25 | Δ*tcpP,* pNL17 (pBAD24, P*_BAD_-tcpP*_C58L_) , P*_toxT_-luxCDABE* | | | | This study |
| NLV26 | Δ*tcpP/*Δ*toxR,* pNL14 (pBAD24, P*_BAD_-tcpP*_C58A_) , P*_toxT_-luxCDABE* | | | | This study |
| NLV27 | Δ*tcpP/*Δ*toxR,* pNL15 (pBAD24, P*_BAD_-tcpP*_C58T_) , P*_toxT_-luxCDABE* | | | | This study |
| NVL28 | Δ*tcpP/*Δ*toxR,* pNL16(pBAD24, P*_BAD_-tcpP*_C58G_) , P*_toxT_-luxCDABE* | | | | This study |
| NLV29 | Δ*tcpP/*Δ*toxR,* pNL17 (pBAD24, P*_BAD_-tcpP*_C58L_) , P*_toxT_-luxCDABE* | | | | This study |
| NLV30 | Δ*tcpP/*Δ*toxR,* pNL3 (pBAD24, P*_BAD_-tcpP*_C58S_) , P*_toxT_-luxCDABE* | | | | This study |
| NLV31 | Δ*tcpP,* pNL18 (pBAD24, P*_BAD_-tcpP*_C19/51/58S_) , P*_toxT_-luxCDABE* | | | | This study |
| NLV32 | Δ*tcpP,* pNL19 (pBAD24, P*_BAD_- tcpP*_C207/218S_-nFLAG) | | | | This study |
| *E.coli* strains | | |  | |  |
| DH5α | | *E. coli* for Cloning strain | | | (Hanahan, 1983) |
| SM10 λ pir | | *E. coli* for conjugation | | | (Yang et al., 2013) |
| BTH101 | | *E. coli* two hybrid system detected strain | | | (Karimova et al., 1998) |
| MSE30 | | DH5α, (pBAD24) , P*_toxT_-luxCDABE* | | | This study |
| MSE35 | | DH5α, pMH501 (pBAD24, P*_BAD_-tcpP*_WT_), P*_toxT_-luxCDABE* | | | This study |
| MSE33 | | DH5α, pNL3 (pBAD24, P*_BAD_-tcpP*_C58S_) , P*_toxT_-luxCDABE* | | | This study |
| MSE34 | | DH5α, pNL14 (pBAD24, P*_BAD_-tcpP*_C58A_) , P*_toxT_-luxCDABE* | | | This study |
| MSE7 | | DH5α, pNL15 (pBAD24, P*_BAD_-tcpP*_C58T_) , P*_toxT_-luxCDABE* | | | This study |
| MSE8 | | DH5α, pNL16 (pBAD24, P*_BAD_-tcpP*_C58G_) , P*_toxT_-luxCDABE* | | | This study |
| MSE9 | | DH5α, pNL17 (pBAD24, P*_BAD_-tcpP*_C58L_) , P*_toxT_-luxCDABE* | | | This study |
| **Cloning** | | | | **Primer Sequence (5’ → 3’)** | |
| P*_BAD_-tcpP*_WT_ | | | | F: GCGGAATTCATGGGGTATGTCCGCGTGAT | |
|  | | | | R: CGGAATTCCTAAAAATCGCTTTGACAGGA | |
| P*_BAD_-tcpP*_C19S_ | | | | F1:GCGGAATTCATGGGGTATGTCCGCGTGAT | |
|  | | | | R1: CTTGATTAGTCGATTCATTCCACC | |
|  | | | | F2: GGTGGAATGAATCGACTAATCAAG | |
|  | | | | R2: CGGAATTCCTAAAAATCGCTTTGACAGGA | |
| P*_BAD_-tcpP*_WT_-nFLAG | | | | F: CGGAATTCATGGATTATAAGGATGATGATGATAAG  GGGTATGTCCGCGTGATTTATCAATT | |
|  | | | | R: CGGAATTCCTAAAAATCGCTTTGACAGGA | |
| P*_BAD_-tcpP*_C58T_ | | | | F1: GCGGAATTCATGGGGTATGTCCGCGTGAT | |
|  | | | | R1: GATCATTTGGAGTGGGGGCAGGATG | |
|  | | | | F2: CATCCTGCCCCCACTCCAAATGATC | |
|  | | | | R2: CGGAATTCCTAAAAATCGCTTTGACAGGA | |
| P*_BAD_-tcpP*_C58L_ | | | | F1: GCGGAATTCATGGGGTATGTCCGCGTGAT | |
|  | | | | R1: GATCATTTGGTAAGGGGGCAGGATG | |
|  | | | | F2: CATCCTGCCCCCTTACCAAATGATC | |
|  | | | | R2: GATCATTTGGTAAGGGGGCAGGATG | |
| P*_BAD_-tcpP*_C58G_ | | | | F1: GCGGAATTCATGGGGTATGTCCGCGTGAT | |
|  | | | | R1: GATCATTTGGACCGGGGGCAGGATG | |
|  | | | | F2: CATCCTGCCCCCGGTCCAAATGATC | |
|  | | | | R2: GATCATTTGGTAAGGGGGCAGGATG | |
| P*_BAD_-tcpP*_C58A_ | | | | F1: GCGGAATTCATGGGGTATGTCCGCGTGAT | |
|  | | | | R1: GATCATTTGGAGCGGGGGCAGGATG | |
|  | | | | F2: CATCCTGCCCCCGCTCCAAATGATC | |
|  | | | | R2: GATCATTTGGTAAGGGGGCAGGATG | |
| P*_BAD_-tcpP*_C58S_ | | | | F1: GCGGAATTCATGGGGTATGTCCGCGTGAT | |
|  | | | | r1: GATCATTTGGTGAGGGGGCAGGATG | |
|  | | | | F2: CATCCTGCCCCCTCACCAAATGATC | |
|  | | | | R2: GATCATTTGGTAAGGGGGCAGGATG | |
| P*_BAD_-tcpP*_C51S_ | | | | F1: GCGGAATTCATGGGGTATGTCCGCGTGAT | |
|  | | | | R1: GGGCAGGATGATATTCTGAGAGTAT | |
|  | | | | F2: TATTGAAAATACTCTCAGAATATCA | |
|  | | | | R2: GATCATTTGGTAAGGGGGCAGGATG | |
| P*_BAD_-tcpP*_C124S_ | | | | F1: GCGGAATTCATGGGGTATGTCCGCGTGAT | |
|  | | | | R1: ATTTTTTTTGACTAGAGTCAGCTTC | |
|  | | | | F2: GTTGTTGATGAAGCTGACTCTAGTCA | |
|  | | | | R2: GATCATTTGGTAAGGGGGCAGGATG | |

**Reference for Supplement figure legends**

Hanahan, D. (1983). Studies on transformation of Escherichia coli with plasmids. *J Mol Biol* 166(4)**,** 557-580.

Joelsson, A., Liu, Z., and Zhu, J. (2006). Genetic and phenotypic diversity of quorum-sensing systems in clinical and environmental isolates of Vibrio cholerae. *Infect Immun* 74(2)**,** 1141-1147. doi: 10.1128/IAI.74.2.1141-1147.2006.

Karimova, G., Pidoux, J., Ullmann, A., and Ladant, D. (1998). A bacterial two-hybrid system based on a reconstituted signal transduction pathway. *Proc Natl Acad Sci U S A* 95(10)**,** 5752-5756.

Miller, J. (1972). in *Experiments in Molecular Genetics*. Cold Spring Harbor Laboratory Press, Cold Spring Harbor, NY).

Yang, M., Liu, Z., Hughes, C., Stern, A.M., Wang, H., Zhong, Z., et al. (2013). Bile salt-induced intermolecular disulfide bond formation activates Vibrio cholerae virulence. *Proc Natl Acad Sci U S A* 110(6)**,** 2348-2353. doi: 10.1073/pnas.1218039110.
